# Transition-Metal-Doped SiP_2_ Monolayer for Effective CO_2_ Capture: A Density Functional Theory Study

**DOI:** 10.1021/acsomega.2c05532

**Published:** 2022-10-07

**Authors:** Kelvin Wang, Xuan Luo

**Affiliations:** National Graphene Research and Development Center, Springfield, Virginia22151, United States

## Abstract

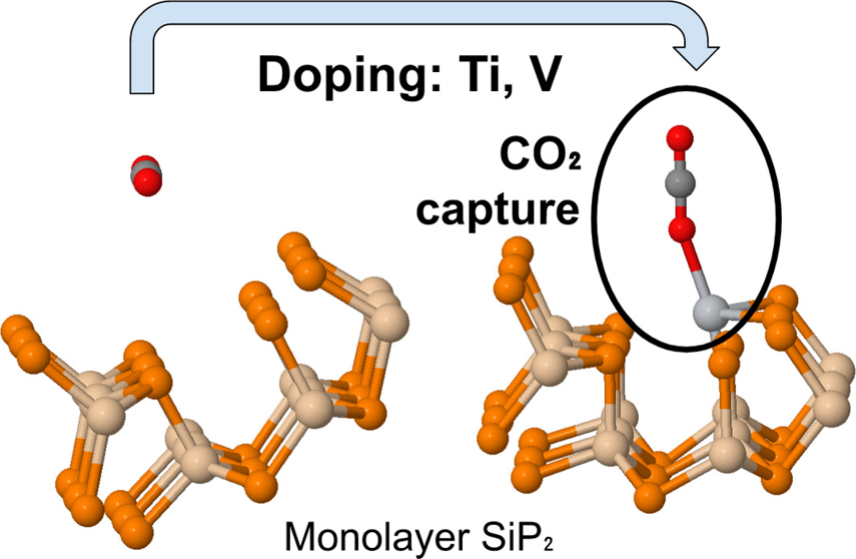

Two-dimensional materials have exhibited great potential in mitigating climate change through sensing and capturing carbon dioxide. The interaction of CO_2_ on orthorhombic silicon diphosphide remains unexplored in spite of its interesting properties such as high carrier mobility, piezoelectricity, and mechanical stability. Here, using density functional theory, the adsorption of CO_2_ on pristine and Ti-, V-, and Cr-doped monolayer SiP_2_ is investigated. Doped systems exhibited significantly stronger adsorption (−0.268 to −0.396 eV) than pristine SiP_2_ (−0.017 to −0.031 eV) and have the possibility of synthesis with low defect formation energies. Our results on adsorption energy, band structure, partial density of states, and charge transfer conclude that titanium- and vanadium-doped SiP_2_ monolayers would be promising materials for CO_2_ capture and removal.

## Introduction

One of the most significant problems countries are facing today is the threat of climate change. Higher sea levels to ocean acidification and global warming have all been documented, where the main cause has been ever-increasing amounts of greenhouse gases in the world since the Industrial Revolution.^1^ In particular, the concentration of carbon dioxide experienced a 46% increase from roughly 280 ppm in the late 1700s to 410 ppm in 2019.^2,3^ Substantial efforts have been made by scientists to mitigate climate change by designing materials capable of capturing carbon dioxide. The most mature method is amine scrubbing, where CO_2_ is absorbed into a liquid solution of monoethanolamine.^4^ The CO_2_–amine solution is then separated under steam, allowing the amine solution to be recycled. However, the process is corrosive, environmentally harmful, and requires a high amount of energy to regenerate the solvent. Therefore, there is a need to search for better methods.^5^

A proposed alternative to the amine process has been to use ionic liquids because they are nonvolatile, stable at high temperatures, and can be tuned to react with CO_2_.^5^ Still, they have not been widely implemented because of their higher price and lower solubility of CO_2_ compared to other solvents. Within the past few decades, there has been a proliferation of interest in using nanomaterials. Nanomaterials possess a naturally active surface and the ability to be functionalized with other materials, which are favorable properties for gas capture. Metal organic frameworks (MOFs) and zeolites, a subfamily of MOFs, have stood out because of their strong sensitivity, tunability, and broad applicability.^6−8^ Amine-functionalized carbon nanotubes have also held great potential because of their high stability and high adsorption capacity.^9^ In recent years, two-dimensional (2D) materials have attracted much attention for CO_2_ capture because of their extremely high surface area–volume ratio and large reaction sites for capture and permeation.^10^

Promising experimental work on 2D materials for gas capturing confirms the value of its continual theoretical research. MXene 2D nanosheets in a mixed-matrix membrane showed good selectivity toward CO_2_ and ran with excellent stability over an extended period of time.^11^ Similar favorable results for 2D ZnAl and Ni–Al layered double hydroxide nanosheets were found as well.^12,13^ A variety of methods, such as doping, could also be used to manipulate the capture. Wang et al. showed that doping graphene with Cu and Ni increased CO_2_ adsorption from weak physisorption to chemisorption.^14^ In another example, platinum-doped silicene improved its attraction to CO_2_.^15^

Over the past few years, monolayer SiP_2_ has attracted much attention over its intriguing properties. Most of the past research focuses on its stability, electronic properties, and potential as a photocatalyst, with limited review of its potential for CO_2_ capture.^16−22^ When CO_2_ was adsorbed on the oblique form of SiP_2_, chemical bonds formed and the structure strongly deformed.^22^ Meanwhile, Yu et al. tested the presence of CO_2_, O_2_, H_2_, N_2_, and H_2_O molecules on SiP_2_ to better understand its stability. Their results revealed that SiP_2_ remained intact, and all the tested gases separated from the substrate.^17^ Notably, there has yet to be research done on the effects of doping to modify the adsorption strength and whether the other SiP_2_ structures are suitable for CO_2_ capture.

In this current research, we used first-principles calculations to search for the most stable atomic structure of the SiP_2_ monolayer. Later, we tested multiple positions to understand the adsorption of CO_2_ on the SiP_2_ monolayer. To improve adsorption, we then doped the SiP_2_ monolayer with titanium, vanadium, and chromium, investigating the band structure, charge transfer, and partial density of states (PDOS).

## Methods

### Computations

We performed first-principles calculations based on density functional theory (DFT) within generalized gradient approximation in the Perdew–Burke–Ernzerhof format^23^ implemented in the ABINIT^24^ suite. For pseudopotentials, we used the projected augmented wave (PAW) method^25^ with projectors generated using ATOMPAW.^26^ The electron configurations used to generate the pseudopotentials are shown in Table 1.

**Table 1:** Electron Configurations and Radius Cutoffs Used to Generate the PAW Pseudopotentials

element	electron configuration	radius cutoffs (a.u.)
silicon (Si)	[Ne]3s^2^3p^2^	1.91
phosphorus (P)	[Ne]3s^2^3p^3^	1.91
carbon (C)	[He]2s^2^2p^2^	1.51
oxygen (O)	[He]2s^2^2p^4^	1.41
titanium (Ti)	[Ne]3s^2^3p^6^4s^1^3d^3^	2.3
vanadium (V)	[Ne]3s^2^3p^6^4s^2^3d^3^	2.2
chromium (Cr)	[Ne]3s^2^3p^6^4s^1^3d^5^	2.1

In the total energy calculation, the self-consistent field (SCF) will be terminated once the total energy difference is smaller than 1.0 × 10^–10^ Ha for the second time. We then performed convergence with the kinetic energy cutoff, Monkhorst–Pack *k*-point grids,^27^ and vacuum. The dataset will be converged when the total energy difference is less than 0.0001 Ha (0.003 eV) twice consecutively. When performing structural relaxations, the SCF cycle will be terminated once the force difference is smaller than 1.0 × 10^–6^ Ha/bohr twice consecutively. The maximum force tolerance in the relaxation is 5.0 × 10^–5^ Ha/bohr.

### Atomic Structure

**Figure 1:**
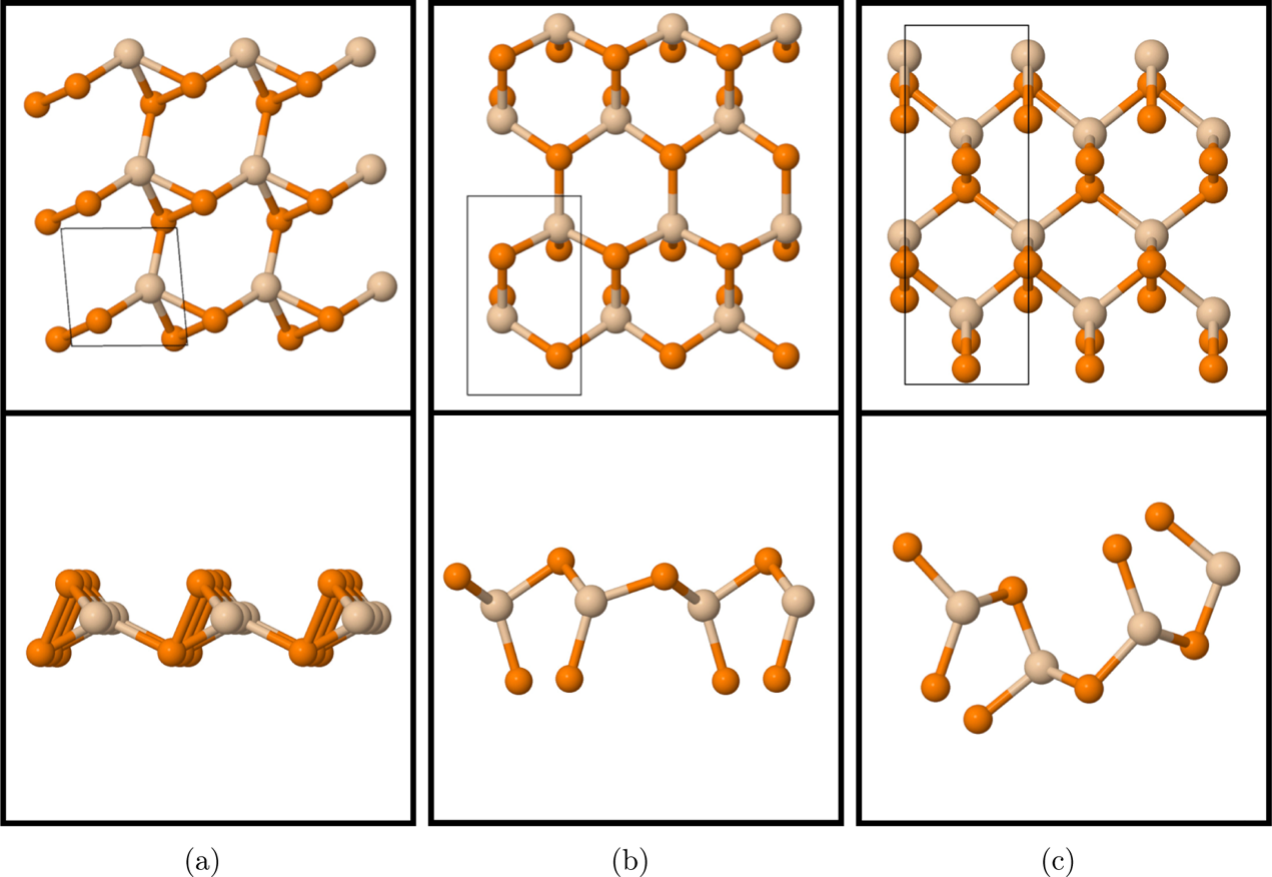
Top view (first row) and side view (second row) of (a) 3 × 3 × 1 oblique SiP_2_, (b) 3 × 2 × 1 Janus SiP_2_, and (c) 3 × 1 × 1 orthorhombic SiP_2_. The frames in the top view represent the unit cells. Tan atoms represent silicon, and orange atoms represent phosphorus.

We examined oblique SiP_2_ because of its CO_2_ capture ability, ultrahigh carrier mobility, and stability.^22^ We also analyzed the Janus structure because it was determined to have the lowest formation enthalpy compared to the other structures.^16^ Orthorhombic SiP_2_ in the *Pbam* phase also captured our attention because it was the most widely researched crystal structure of SiP_2_ and had a lower energy compared to the tetragonal structure.^18−21,28^ We converged and relaxed the structures using the 3-atom unit cell for oblique, 6-atom cell for Janus, and 12-atom cell for orthorhombic SiP_2_ (Figure 1). Calculated lattice constants are shown in (Table 2).

**Figure 2:**
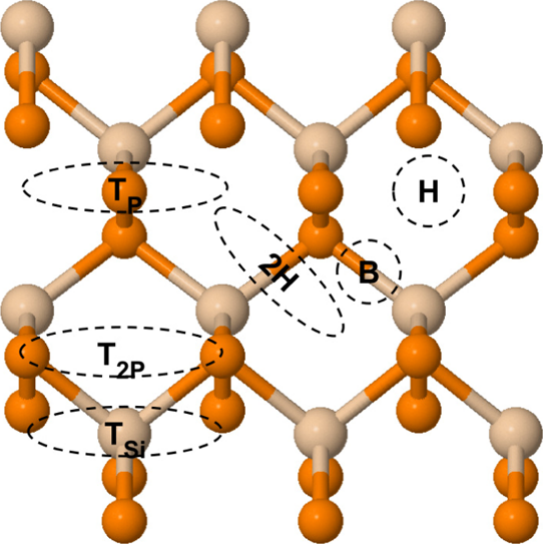
Relaxed structure of 3 × 1 × 1 SiP_2_ and six possible adsorption sites of CO_2_ we analyzed. T denotes that CO_2_ was placed at the top of an atom, H denotes the placement at the hollow center of the upper hexagon, and B denotes that CO_2_ was placed at the bond. Oval shapes represent where CO_2_ was placed horizontally and circles represent where CO_2_ was placed vertically. Tan and orange atoms denote silicon and phosphorus, respectively.

After analyzing the pristine SiP_2_ monolayers, we used orthorhombic SiP_2_ for CO_2_ adsorption. We placed the fully relaxed CO_2_ molecule on the 3 × 1 × 1 SiP_2_ monolayer at six typical positions, as shown in Figure 2. CO_2_ is parallel to the surface in T_Si_ (on top of a silicon atom), T_P_ (on top of a phosphorus atom), T_2P_ (between two phosphorus atoms in the upper hexagon), and 2H site (on top of the Si–P bond between the lower and upper hexagons). At H (on top of the upper hexagon) and B (on top of the Si–P bond), CO_2_ is placed vertically to the surface. To calculate the adsorption energies of the various positions, we defined it as
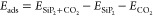
1where *E*_SiP_2_+CO_2__ is the total energy of the SiP_2_ monolayer adsorbed with CO_2_, *E*_SiP_2__ is that of the pristine monolayer, and *E*_CO_2__ is that of a single CO_2_ molecule. A negative *E*_ads_ denotes that CO_2_ can be adsorbed on the monolayer and a more negative *E*_ads_ denotes stronger adsorption. Later, when we substituted titanium, vanadium, and chromium for phosphorus, we calculated defect formation energy as
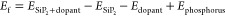
2where *E*_SiP_2_+dopant_, *E*_SiP_2__, *E*_dopant_, and *E*_phosphorus_ represent the total energy of doped SiP_2_, pure SiP_2_, and the chemical potential for Ti, V, and Cr dopant and the removed *E*_phosphorus_ atom, respectively.

To understand the charge transfer in the doped systems, we used
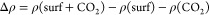
3where ρ(surf + CO_2_), ρ(surf), and ρ(CO_2_) are the charge density of the CO_2_–SiP_2_ system, SiP_2_ monolayer, and CO_2_ molecule, respectively.

To plot all the band structures, we used the high-symmetry *k*-points along Γ(0, 0, 0), *Y*(0.5, 0, 0), Σ(0.5, 0.5, 0), *X*(0, 0.5, 0), and Σ(0, 0, 0).

## Results and Discussion

### Outline

We first analyze the structural and electronic properties of pristine monolayer SiP_2_. Next, we examine the adsorption of CO_2_ on pristine monolayer SiP_2_. Afterward, we discuss the interactions between doped SiP_2_ and CO_2_ using the band structure, PDOS, and charge transfer.

**Table 2:** Calculated Lattice Constants of the Three Identified SiP_2_ Structures and the Theoretical and Experimental Lattice Constants Previously Obtained by Other Researchers^a^

		calculated	others	bulk exp	% error
oblique	*a* (Å)	3.73	3.71^22^		
	*b* (Å)	3.73	3.74		
Janus	*a* (Å)	3.45	3.45^17^		
	*b* (Å)	6.04	6.05		
orthorhombic	*a* (Å)	3.47	3.44^20^	3.44^29^	0.86
	*b* (Å)	10.00	10.00	10.08	0.8
	*c* (Å)			13.97	

a Percent error is determined by calculated and bulk experimental values.

### Pristine SiP_2_

**Figure 3:**
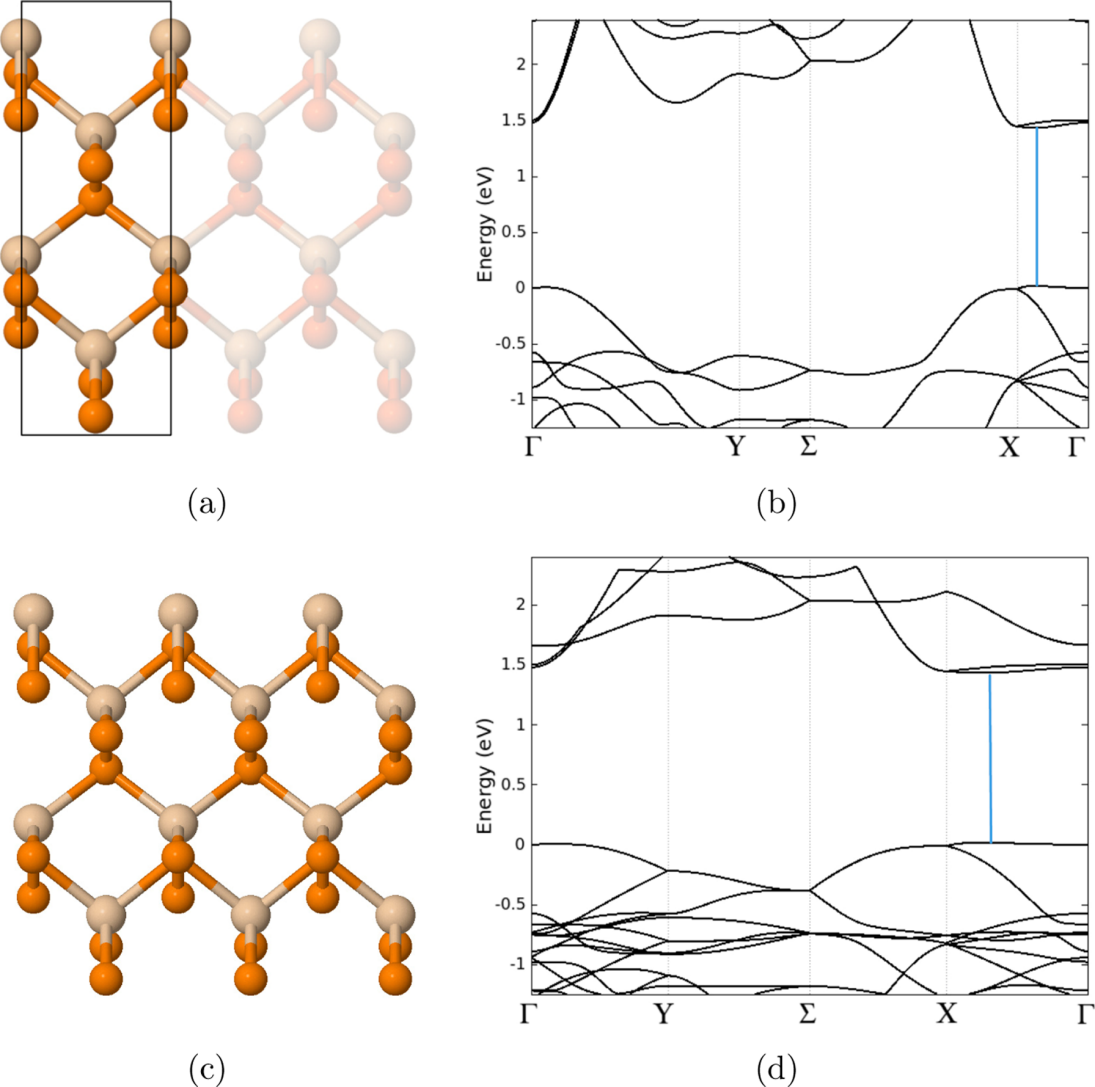
Atomic structure and band structure of the orthorhombic SiP_2_ monolayer. (a) Shows the 1 × 1 × 1 unit cell of SiP_2_, (b) band structure of 1 × 1 × 1 SiP_2_, (c) 3 × 1 × 1 cell of SiP_2_, and (d) band structure of 3 × 1 × 1 SiP_2_. Both structures have a direct band gap of 1.41 eV, as denoted by the blue line. The Fermi level has been set to 0 eV for both graphs. Tan atoms denote silicon, and orange atoms denote phosphorus.

We determined that the orthorhombic structure is energetically more stable than the oblique and Janus structure by −0.895 and −0.003 eV, respectively. Figure 3b,d shows that the unit cell and 3 × 1 × 1 orthorhombic monolayer SiP_2_ are both semiconductors with a direct band gap of 1.41 eV, and the conduction band minimum and valence band maximum are in the *X*–Γ path. Although the DFT method is known to underestimate the band gap, the general trend in the unit cell band structure agrees with previously reported findings.^20,21^

### CO_2_ Adsorption on Pristine SiP_2_

**Figure 4:**
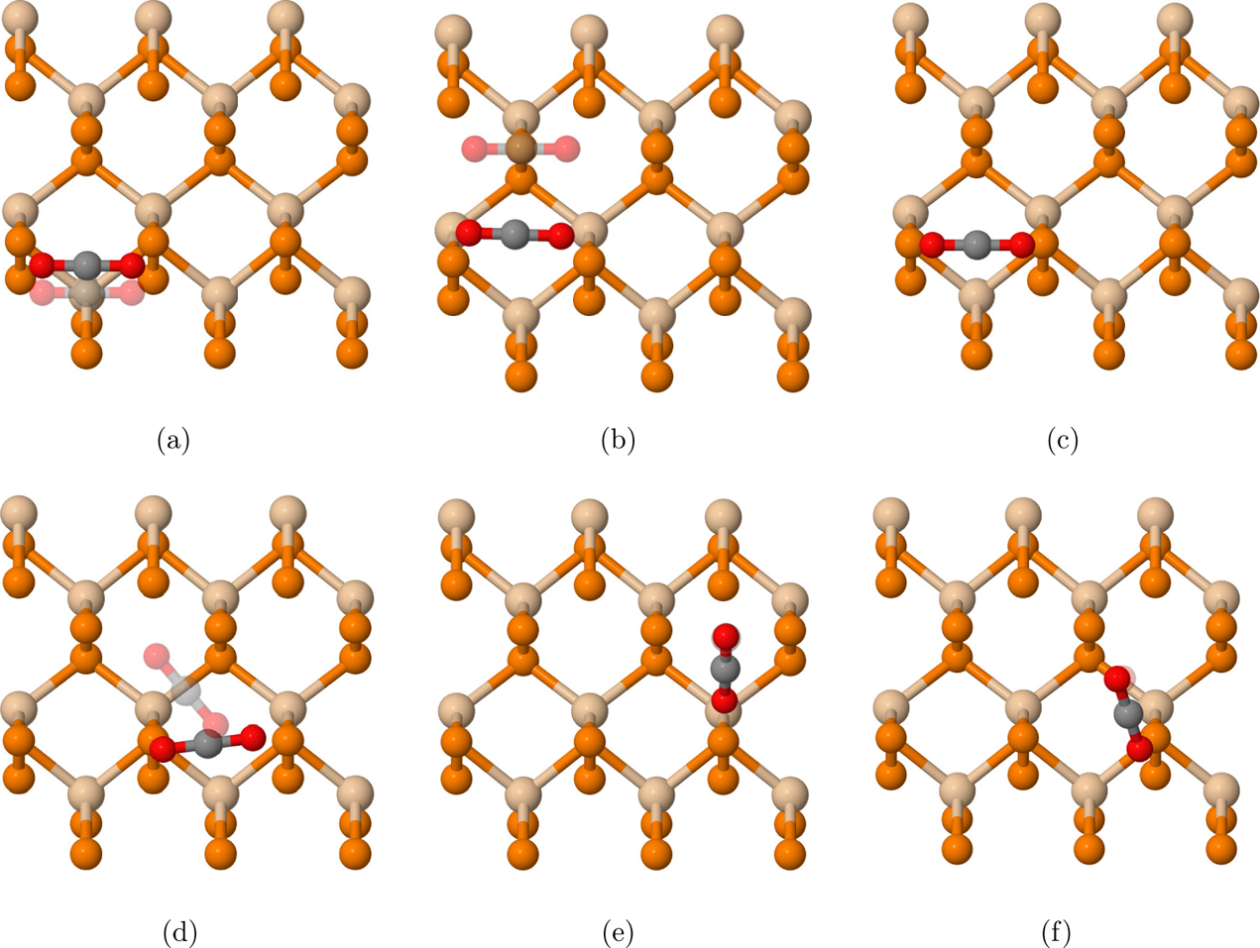
Top view of CO_2_ relaxed at (a) T_Si_, (b) T_P_, (c) T_2P_, (d) 2H, (e) H, and (f) B in monolayer SiP_2_. Transparent and shaded CO_2_ denotes the original and final positions. Tan, orange, red, and gray atoms denote silicon, phosphorus, oxygen, and carbon, respectively.

The corresponding adsorption energies, vertical distance of CO_2_ to the substrate, and smallest atom–atom distance between CO_2_ and SiP_2_ are listed in Table 3. Corresponding structures are shown in Figure 4.

**Table 3:** Vertical Distance between CO_2_ and the Substrate Is Represented by *d*_vertical_, the Smallest Atom-to-Atom Distance between the Substrate and CO_2_ Is Denoted by *d*_atom–atom_, and the Adsorption Energy of CO_2_ Is Denoted by *E*_ads_

	T_Si_	T_P_	T_2P_	2H	H	B
*d*_vertical_ (Å)	2.20	2.39	1.98	2.03	2.43	1.86
*d*_atom–atom_ (Å)	3.83	3.99	4.04	4.12	3.86	3.96
*E*_ads_ (eV)	–0.028	–0.023	–0.031	–0.029	–0.017	–0.017

As seen in Figure 4a, carbon moved away from T_Si_ to the center of the lower hexagon with a distance of 3.83 Å to the nearest molecule. When CO_2_ was initially placed at T_P_, it also moved to the center of the lower hexagon, which can be seen upon comparing the before and after positions in Figure 4b. The energetically most favorable site had an adsorption energy of −0.031 eV at T_2P_, where CO_2_ keeps its initial horizontal position in Figure 4c. In Figure 4d, O rotated from 2H to the center of the lower hexagon with both C atoms near opposite P atoms. The energetically least favorable site occurred at H with an adsorption energy of −0.017 eV (Figure 4e). The O atom closest to the monolayer tilted away from the upper hexagon toward Si, while the other O atom remained at H. When placed vertically at B in Figure 4f, CO_2_ tilted toward the substrate and shifted to the edge of the lower hexagon.

In Table 3, we see how a smaller vertical distance does not correlate with a smaller atom–atom distance and adsorption energy. 2H has the largest atom–atom distance of 4.12 Å; however, its vertical distance is smaller than other positions. T_2P_ has a larger atom–atom distance than most other positions but the highest adsorption energy. Meanwhile, B has the smallest vertical distance of 1.86 Å, while its adsorption energy is closest to zero. Comparing the positions shown in Figure 4 with the adsorption energy, we find that CO_2_ prefers to adsorb parallel at the center of the lower hexagon. As all the adsorption energies are small and there is a large distance from the monolayer at all the positions, pure monolayer SiP_2_ is ineffective for CO_2_ capture.

### Ti-, V-, and Cr-Doped Monolayer SiP_2_

Finding that the adsorption energies on the pristine SiP_2_ monolayer were weak, we substituted Ti, V, and Cr for phosphorus. Doping Ti, V, and Cr resulted in strong adsorption energies of CO_2_ compared to other dopants across multiple monolayers.^30−34^ Other dopants such as Mn and Fe did also exhibit strong adsorption; however, the strength of their adsorption was not as consistent as Ti, V, and Cr. Since titanium has four valence electrons, 4s^1^3d^3^, and carbon is an electron acceptor with four valence electrons given by 2s^2^2p^2^, we predicted that Ti-doped SiP_2_ could adsorb CO_2_ through strong ionic bonding between Ti and C. We used the T_P_ position for substitutional doping as phosphorus is closest to the surface, and favorable results were found in past research by doping group IV/V elements.^35−39^

We obtained low defect formation energies of 1.288, 1.835, and 1.002 eV for Ti-, V-, and Cr-SiP_2_, respectively, by using eq 2 outlined in Methods. With chromium-doped SiP_2_, its five electrons in its outer 3d shell can pair with the five valence electrons of phosphorus to form a full 3d orbital, allowing Cr to interact stably with the nearby phosphorus atoms, and support the calculated lowest defect formation energy.

**Figure 5:**
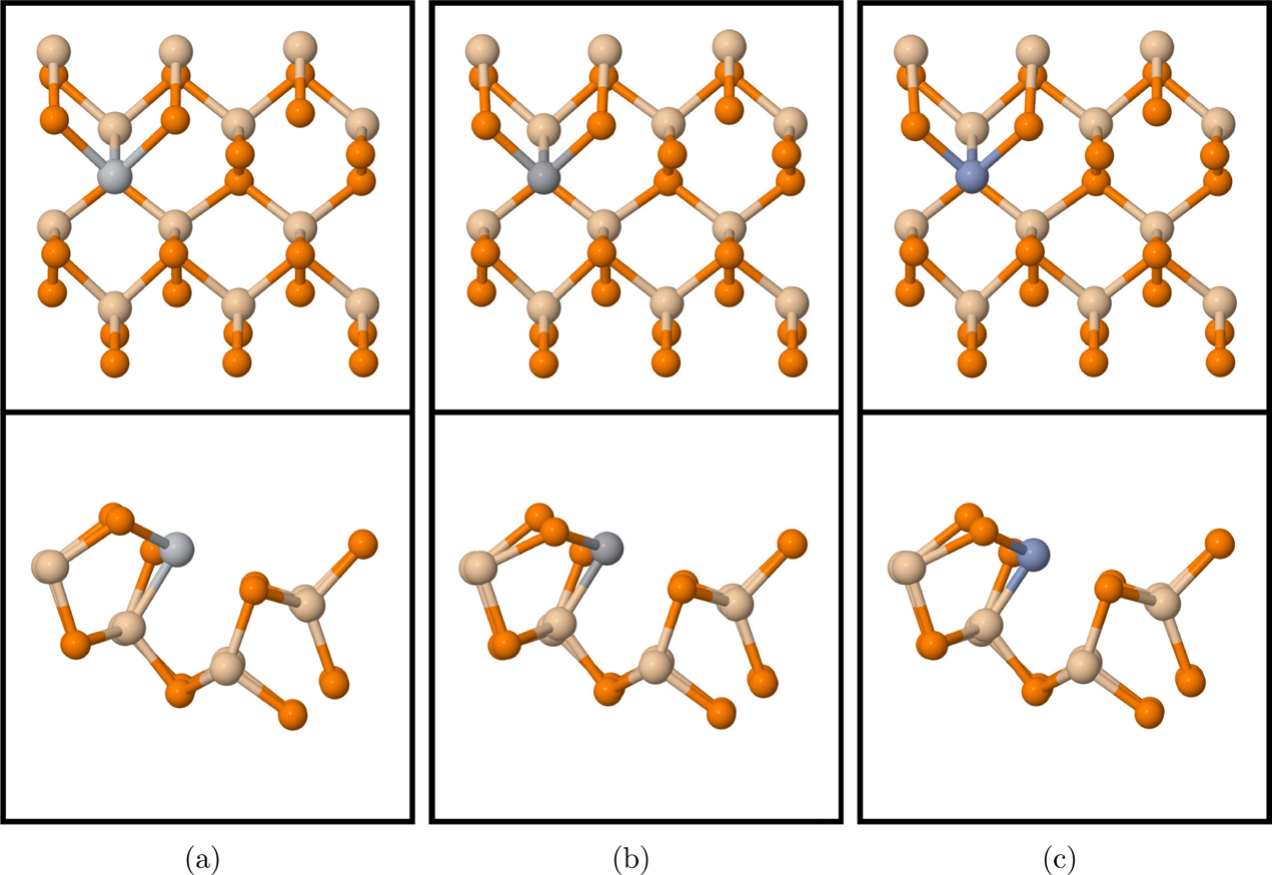
Top view (first row) and side view (second row) of transition metal (a) Ti-, (b) V-, and (c) Cr-doped SiP_2_ monolayers. Tan, orange, and gray/blue atoms denote silicon, phosphorus, and the doped Ti, V, and Cr atom.

**Figure 6:**
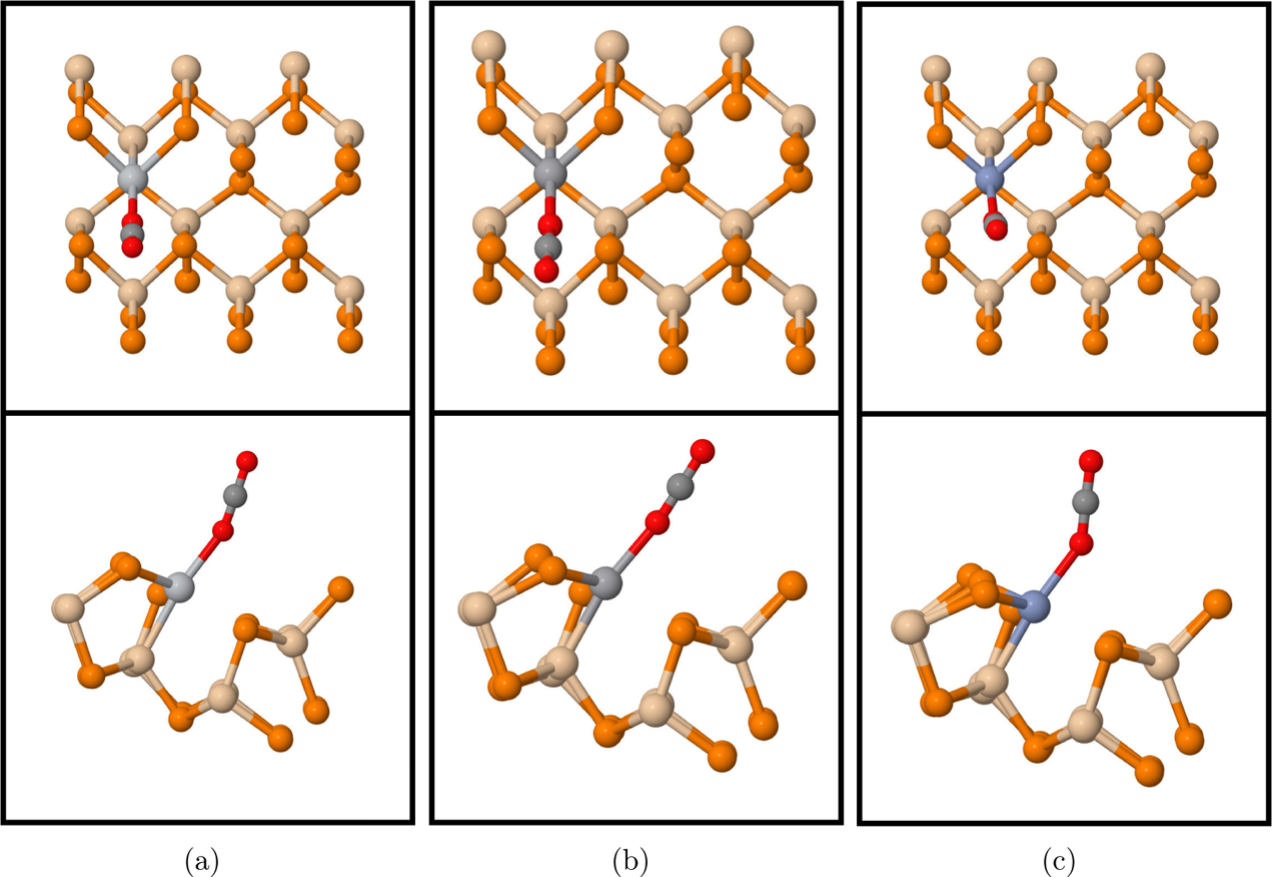
Top view (first row) and side view (second row) of CO_2_ adsorbed on (a) Ti-SiP_2_, (b) V-SiP_2_, and (c) Cr-SiP_2_ monolayers. Tan, orange, red, dark-gray, and light-gray/blue atoms denote silicon, phosphorus, oxygen, carbon, and Ti, V, or Cr, respectively.

After structural relaxation of Ti-doped monolayer SiP_2_, Ti moves to the top of P, expanding the Ti–P–Si angle from 98.8 to 110.8°, as can be seen in the side view of Figure 5a. As can be seen in the side view of Figure 5b, the V–P–Si angle becomes 122.1° and the V–Si distance elongates from 2.34 to 2.48 Å. As shown in Figure 6c, Cr prefers to move above phosphorus with a Cr–P–Si angle of 118.1° and a P–Cr–P angle of 91.6°.

### CO_2_ Adsorption on Ti-, V-, and Cr-Doped Monolayer SiP_2_

The adsorption energies, vertical distance, and smallest atom–atom distance between the doped substrates and CO_2_ are shown in Table 4.

**Table 4:** Vertical Distance between CO_2_ and the Doped SiP_2_ Substrates Is Represented by *d*_vertical_, the Smallest Atom-to-Atom Distance between the Substrates and CO_2_ Is Denoted by *d*_atom–atom_, and the Adsorption Energy of CO_2_ Is Denoted by *E*_ads_

	Ti-SiP_2_	V-SiP_2_	Cr-SiP_2_
*d*_vertical_ (Å)	1.08	0.99	0.95
*d*_atom–atom_ (Å)	2.28	2.22	2.24
*E*_ads_ (eV)	–0.396	–0.363	–0.268

The adsorption energies of CO_2_ on the Ti-, V-, and Cr-SiP_2_ monolayer are calculated to be −0.396, −0.363, and −0.268 eV, respectively. In all the systems, CO_2_ prefers to adsorb vertically above the lower hexagon and form a bond between the dopant and O in the range of 2.22 to 2.28 Å (Figure 6).

**Figure 7:**
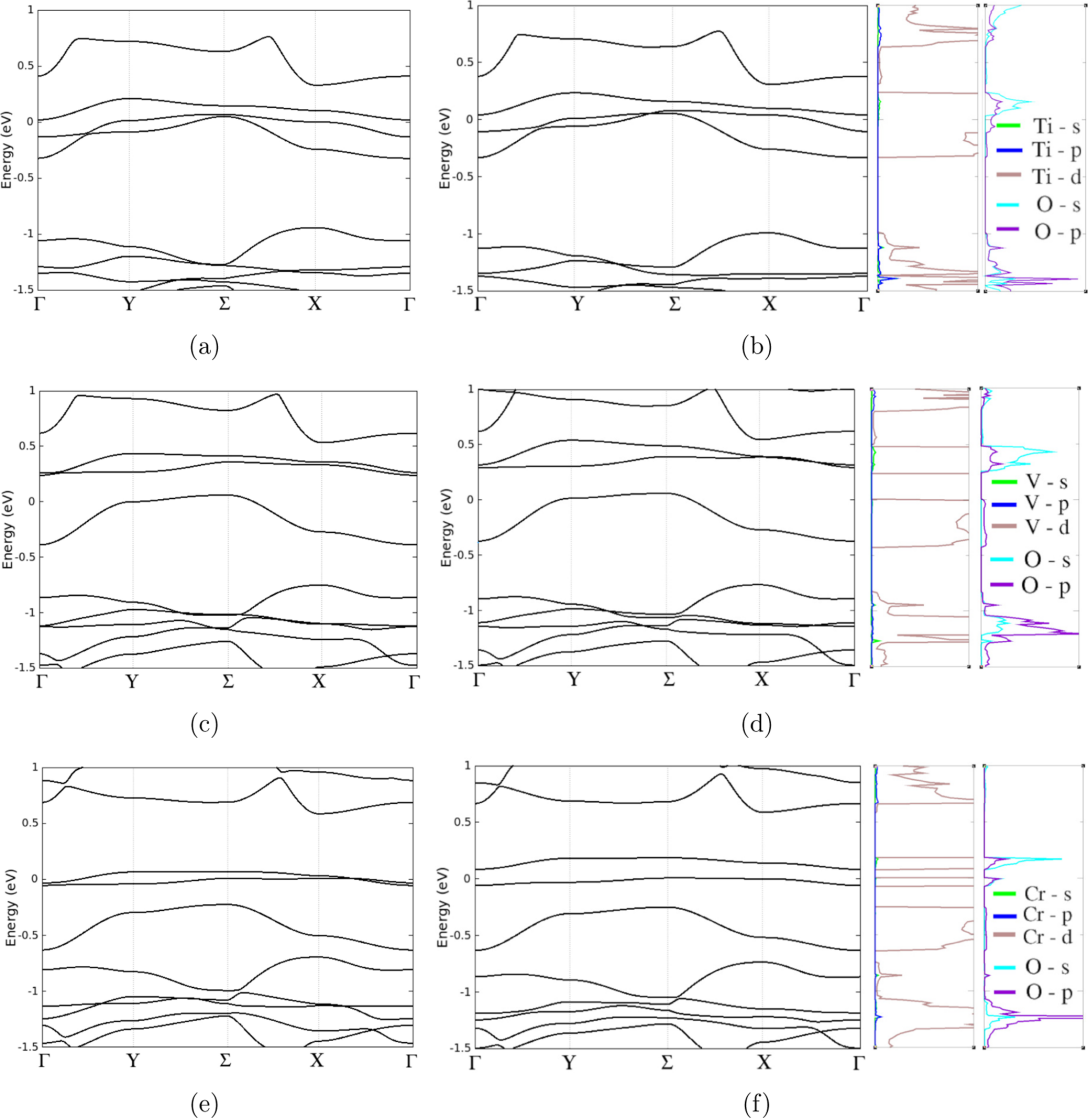
The band structures of (a) Ti-doped SiP_2_, (b) Ti-doped SiP_2_ with CO_2_, (c) V-doped SiP_2_, (d) V-doped SiP_2_ with CO_2_, (e) Cr-doped SiP_2_, and (f) Cr-doped SiP_2_ with CO_2_. PDOS for Ti-doped SiP_2_, V-doped SiP_2_, and Cr-doped SiP_2_ after CO_2_ adsorption have been plotted next to their corresponding band structures. The Fermi level has been set to 0 eV.

With transition-metal doping of SiP_2_, we found that the system became metallic and the adsorption of CO_2_ barely affected the band structure (Figure 7). Figure 7 also presents the PDOS. When CO_2_ was adsorbed on Ti-SiP_2_, strong hybridization was present between the O s, p and Ti d orbitals from −1.5 to −1 eV and weak hybridization between 0.75 and 1 eV. On V-SiP_2_, hybridization occurred between O s, p and V d from −1.5 to −0.8 eV and from 0.3 to 0.5 eV. The O s, p and Cr d orbitals in the CO_2_/Cr-SiP_2_ system hybridized from −1.5 to −0.8 eV and from −0.1 to 0.2 eV.

**Figure 8:**
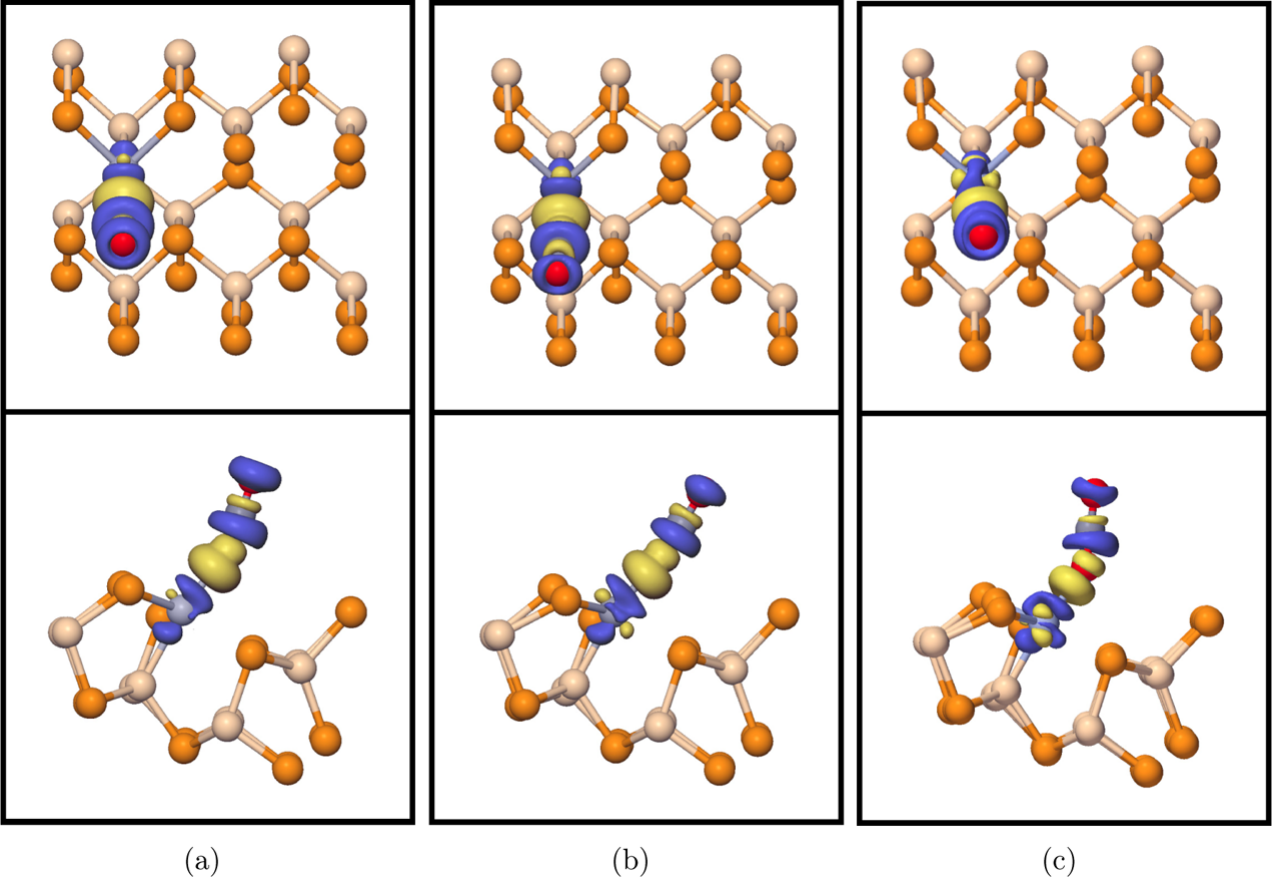
Top view (first row) and side view (second row) of charge transfer between CO_2_ and (a) Ti-SiP_2_, (b) V-SiP_2_, and (c) Cr-SiP_2_. Yellow and blue isosurfaces denote charge accumulation and depletion, respectively.

Next, we plotted the charge transfer (Figure 8) using eq 3 outlined in Methods. Yellow regions represent charge accumulation, and blue regions denote charge depletion. Given that oxygen is more electronegative than the transition-metal dopants, it can be seen that charge depletes from the dopant and accumulates at O. The larger areas of charge transfer in Ti-SiP_2_ and V-SiP_2_ compared to Cr-SiP_2_ correspond to their higher adsorption energies.

## Conclusions

In summary, we used DFT to study the adsorption of CO_2_ on orthorhombic SiP_2_. Various adsorption sites were considered on pristine SiP_2_, finding small adsorption energies and large adsorption distances unsuitable for gas capture. However, doped Ti-, V-, and Cr-SiP_2_ systems showed significantly higher adsorption energies and their low defect formation energies suggest the feasibility of their synthesis. Band structure, PDOS, and charge transfer calculations were also performed, confirming that CO_2_ was strongly adsorbed. Our findings show that doped Ti- and V-SiP_2_ hold promise to be used in temperature swing adsorption for CO_2_ capture from flue gas.^40^ Further research should analyze the total adsorption capacity and working capacity of Ti- and V-SiP_2_ to understand their energy penalty and purity of CO_2_ capture.
